# Dense bone islands in pediatric patients: a case series study

**DOI:** 10.1007/s40368-020-00596-w

**Published:** 2021-01-10

**Authors:** S. Alfahad, M. Alostad, S. Dunkley, P. Anand, S. Harvey, J. Monteiro

**Affiliations:** 1 Department of Paediatric Dentistry, RNENT and Eastman Dental Hospital, 47-49 Huntley St, Bloomsbury, London, WC1E 6DG UK; 2 Department of Radiology, RNENT and Eastman Dental Hospital, 47-49 Huntley St, Bloomsbury, London, WC1E 6DG UK; 3 Great Ormond Street Hospital (Previously: RNENT and Eastman Dental Hospital), Great Ormond Street, London, WC1N 3JH UK

**Keywords:** Lesions, Bony, Pathology, Radiology

## Abstract

**Background:**

Dense Bone Islands (DBIs) are anatomic variants defined as radiopaque lesions consisting of hamartomatous cortical bone, often presenting as incidental radiographic findings. DBIs can also be known as idiopathic osteosclerosis, bone whorl, focal periapical osteopetrosis, bone scar and enostosis. We found a paucity of literature for management and reporting of this condition in children. For this reason, the authors describe sixteen cases of children and adolescents with dense bony islands and suggest a pathway for management.

**Case series:**

Cases presented to the RNENT and Eastman Dental Hospital or private practice, either as chance findings or for diagnosis and treatment planning of undiagnosed radiopaque areas. The individuals were aged between 10 and 17 years; 6 boys and 10 girls. All radiographic reports described DBIs. Diagnoses were confirmed by a Dental and Maxillofacial Radiology Consultant and advised no intervention. In some cases, monitoring was advised. Caution in orthodontic tooth movement was advised for five patients.

**Conclusion:**

DBIs are common findings that seldom require treatment; however, caution should be exercised when undertaking orthodontic movement in the area of a DBI due to a potential risk of root resorption. Accurate identification and multidisciplinary management are of utmost importance.

## Introduction

Dense Bone Islands (DBIs) are anatomic variants defined as radiopaque lesions consisting of hamartomatous cortical bone, often presenting as incidental radiographic findings. DBIs can also be known as idiopathic osteosclerosis, bone whorl, focal periapical osteopetrosis, bone scar and enostosis (Yonetsu et al. 1997). These lesions are thought to be the result of failure of resorption during endochondral ossification and are thought to be congenital or developmental (Diab et al. 2014). Histologically, DBIs can be described as dense calcified tissue without marrow space or inflammatory cell infiltration (Eversole et al. 1984). The lesions can be found in the whole body, with preference for long bones. They are more common in adults, with no gender preference. Dense bone islands of the jaws are considered idiopathic, typically present as asymptomatic incidental findings, with no intra-oral manifestations and no bony expansion. DBIs may vary in size, outline, shape and density. Prevalence of DBIs ranges between 1.7 and 5.4%, with the mandibular molar and premolar regions more frequently affected (Marmary and Kutiner 1986; Petrikowski and Peters 1997). The lesion may increase in size and may complicate orthodontic treatment or implant placement. Differential diagnosis includes periapical cemental dysplasia, osteoma, complex odontoma, cementoblastoma, osteoblastoma and hypercementosis (McDonnell 1993).

We found a paucity of literature for management and reporting of this condition in children. For this reason, the authors describe sixteen cases of children and adolescents with dense bony islands and suggest a pathway for management.

## Case series

Cases presented to the RNENT and Eastman Dental Hospital or private practice, either as chance findings or for diagnosis and treatment planning of undiagnosed radiopaque areas. The individuals were aged between 10 and 17 years; 6 boys and 10 girls. All radiographic reports described DBIs (Table 1).

**Table 1:** Summary of patients DBI characteristics

Case	Age	Gender	Location	Image type	Shape and size
1	11	Male	Bilateral, maxillary right and left premolar regions, between apices of UR4 and UR5; UL5 and UL6	IOPA	From apices of UR4 and UR5 to the floor of maxillary sinus; approximately 8–10 mm
2	14	Female	Unilateral mandibular left quadrant, between roots of LL4 and LL6	IOPA	13 mm, rounded
3	17	Female	Unilateral mandibular left quadrant; between roots of LL5 and LL4	IOPA	7 × 6 mm, attached to the inner aspect of the lingual cortical plate, passing into the medullary cavity with approximately 6.5 mm to the root of LL4 and separated from the root of LL5 by approximately 2.6 mm
4	15	Male	Unilateral mandibular between LL4 and LL5 and apical to LL3	DPT	Well defined, between LL4 and LL5: 9 × 10 mm
5	16	Male	Unilateral mandibular between the apices of LL4 and LL5	CBCT	Irregular and heterogeneous, 9 × 10 × 13 mm
6	15	Female	Unilateral mandibular surrounding apex of LL4	DPT	Round shape, approximately 5 mm diameter
7	14	Female	Unilateral mandibular between the apices of LL6	DPT	5 mm in diameter
8	14	Female	Unilateral mandibular between apices of LL5 and LL6	DPT	Well defined
9	13	Female	Unilateral mandibular mesial to the roots of LL6	DPT	Small rounded
10	17	Male	Unilateral mandibular, mesial to the roots of LR6	DPT	Ovoid, roughly 4 mm × 11 mm
11	12	Male	Unilateral mandibular distal to the apex of LR6	DPT	Oval shaped, well defined
12	17	Female	Unilateral mandibular apical to LR5	DPT	Round, well defined; approximately 15 mm in diameter
13	10	Female	Unilateral mandibular mesial to the root of LR5	DPT	Oval shaped
14	14	Male	Unilateral mandibular apical to apex of LR4	DPT	Oval, well defined
15	15	Female	Unilateral mandibular, from midline to the LL4 apical region	DPT	20 mm length × 20 mm height
16	16	Female	Unilateral, maxillary between UR3 and UR2	DPT and CBCT	Extensive. 11 mm width × 7 mm depth × 24 mm height

*IOPA* intra-oral periapical radiograph, *CBCT* cone beam computed tomography, *DPT* dental panoramic tomograph

**Fig. 1:**
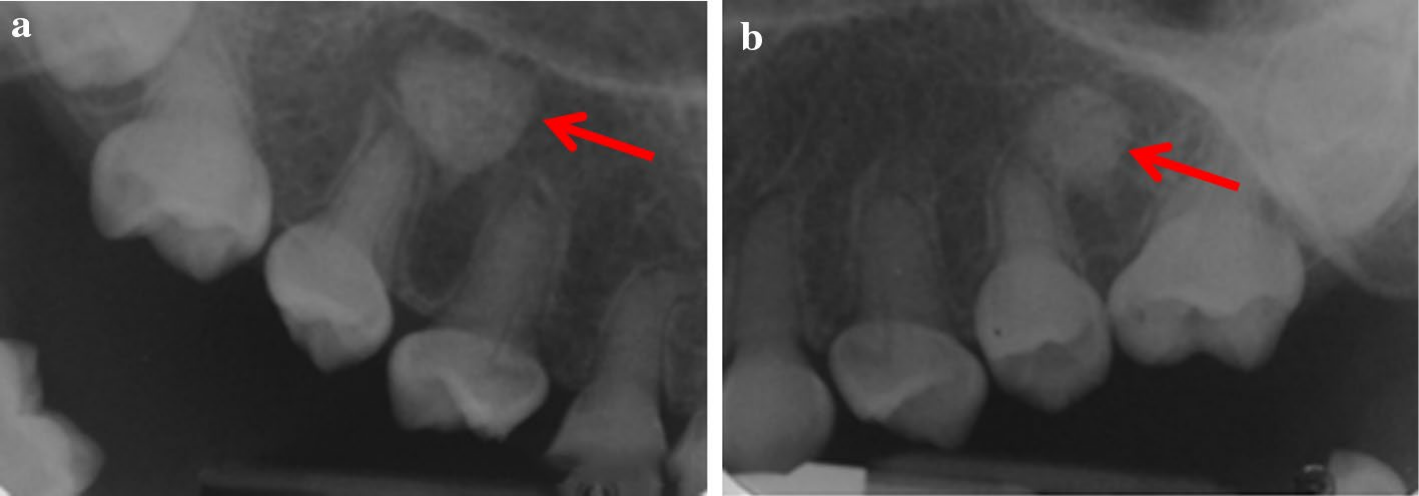
**a**, **b** Periapical radiographs of case 1 showing two DBIs (see arrows) in the maxillary premolar regions. Anatomy of the teeth is typical of dentinogenesis imperfecta type II

**Fig. 2:**
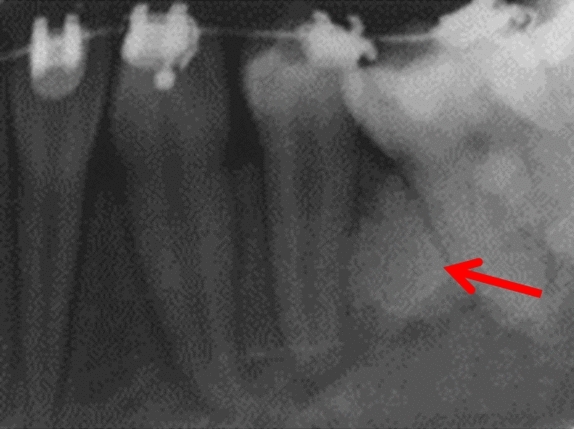
Periapical radiographs of cases 2

**Fig. 3:**
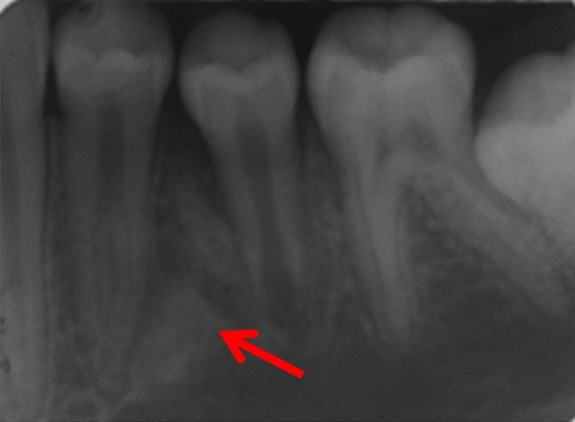
Periapical radiographs of cases 3 showing DBIs (see arrows) in the mandibular left quadrant, in close proximity with the roots of the adjacent teeth

**Fig. 4:**
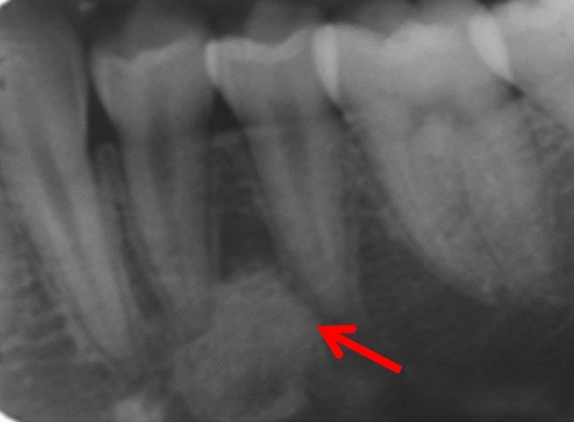
Periapical radiographs of cases 4 showing DBIs (see arrows) in the mandibular left quadrant, in close proximity with the roots of the adjacent teeth

Most cases were unilateral and located in the body of mandible: Five in the lower right quadrant (Fig. 4) and seven in the lower left quadrant (Figs. 2 and 3). One case had a DBI in the maxillary alveolus (Fig. 1). DBIs measured between 1.5 and 13 mm. Interestingly, one patient (case 1) had a diagnosis of dentinogenesis imperfecta, another patient was in remission for acute lymphoblastic leukemia, while the other patients were referred to specialist care for caries. None of the patients were symptomatic at assessment (Figs. 2, 3 and 4).

**Fig. 5:**
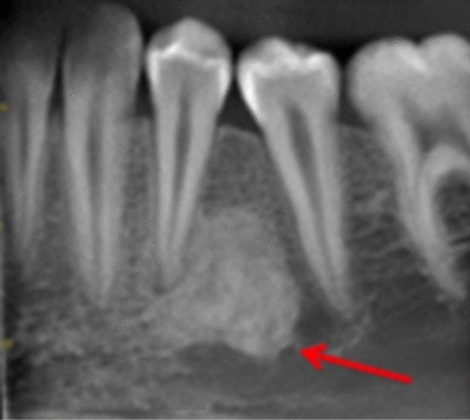
(case 5): Section of the cone beam computed tomography showing a heterogenous DBI (see arrow) around and distal to the root of LR4

**Fig. 6:**
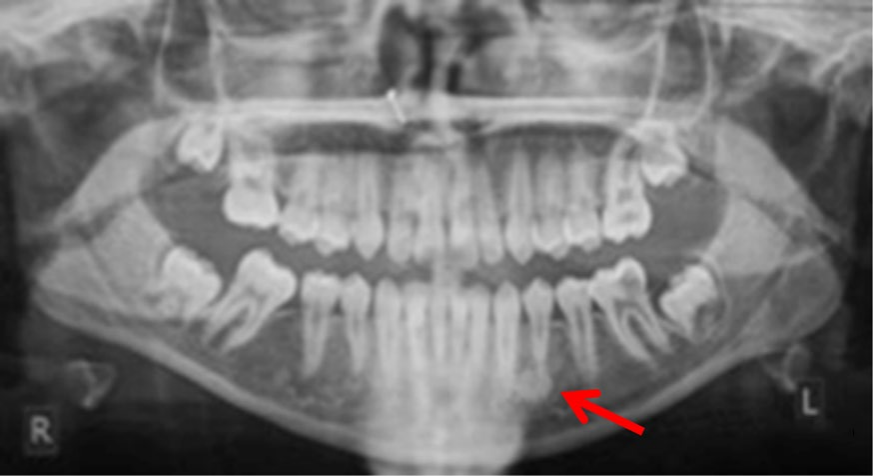
Dental panoramic tomographs showing DBIs (see arrows) in the mandibular left quadrants, apically to LL4 (case 6)

**Fig. 7:**
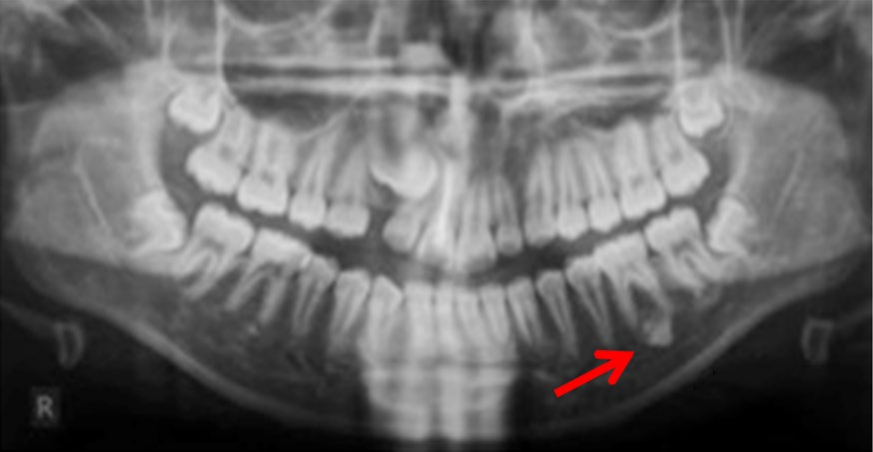
Dental panoramic tomographs showing DBIs (see arrows) in the mandibular left quadrants, apical to LL6 (case 7)

**Fig. 8:**
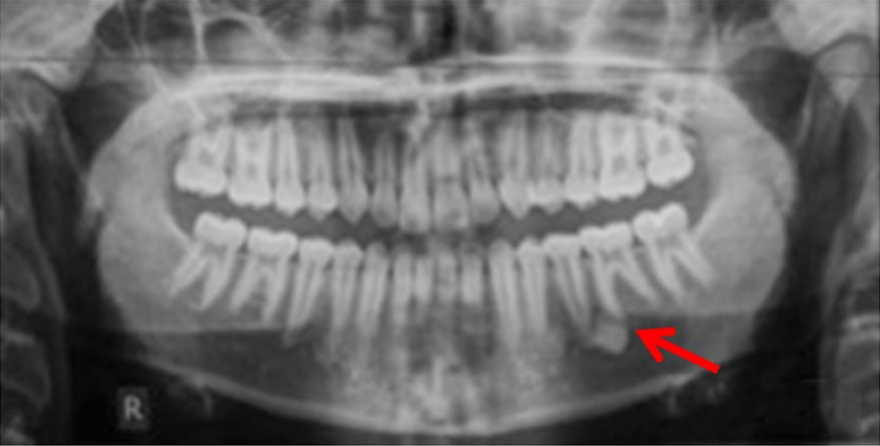
Dental panoramic tomographs showing DBIs (see arrows) in the mandibular left quadrants, apical to LL5 and LL6 (case 8)

**Fig. 9:**
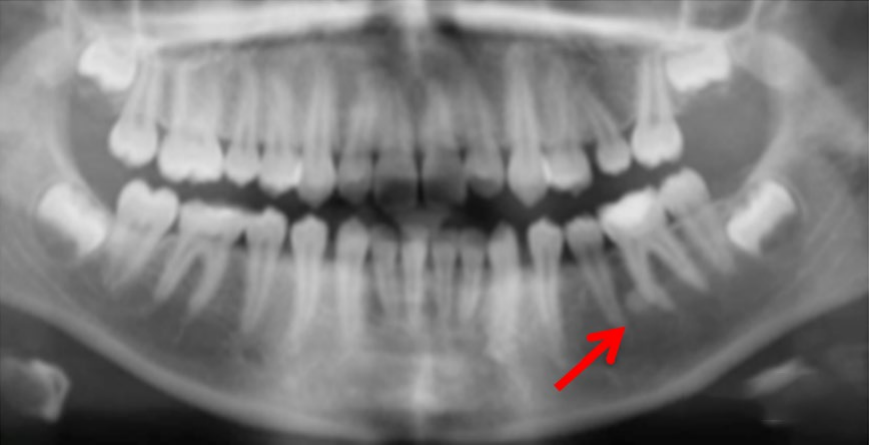
Dental panoramic tomographs showing DBIs (see arrows) in the mandibular left quadrants, mesial to LL6 (case 9)

Three patients were referred by an orthodontist or a general dental practitioner, prior to orthodontic treatment (cases 3, 9 and 12). Two patients had ongoing (case 2) or completed orthodontic treatment (case 4). Six cases were chance radiographic findings (cases 6, 7, 9, 11, 13 and 14) and three cases were referred for management of the radiopaque areas (cases 1, 5 and 8). None of the cases had evidence of resorption of adjacent teeth (Figs. 5, 6, 7, 8 and 9).

**Fig. 10:**
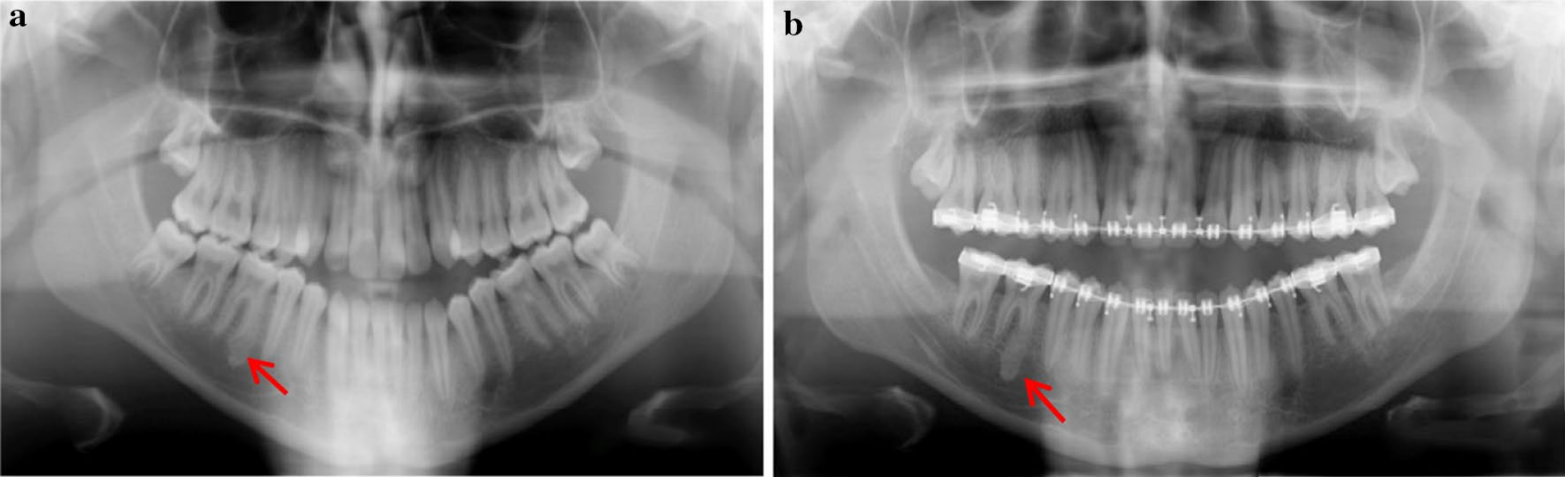
**a**, **b** Dental panoramic tomographs showing a DBI (see arrows) on the mandibular right quadrant, mesially to LR6. DPT taken prior to orthodontic treatment (**a**/case 10) and during orthodontic treatment (**b**/case 10), the latter showing resorption of the mesial root of LR6

**Fig. 11:**
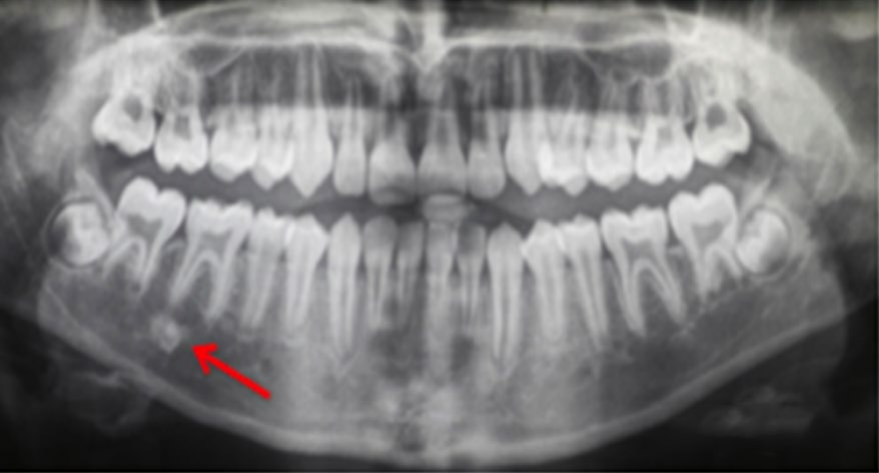
Dental panoramic tomographs showing DBIs (see arrows) on the mandibular right quadrants, apical to LR6 (case 11)

**Fig. 12:**
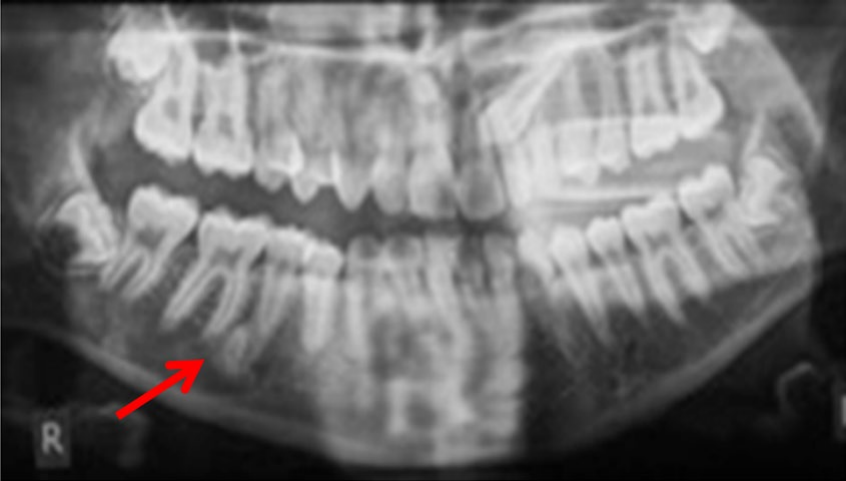
Dental panoramic tomographs showing DBIs (see arrows) on the mandibular right quadrants, distal to LR5 (case 12)

**Fig. 13:**
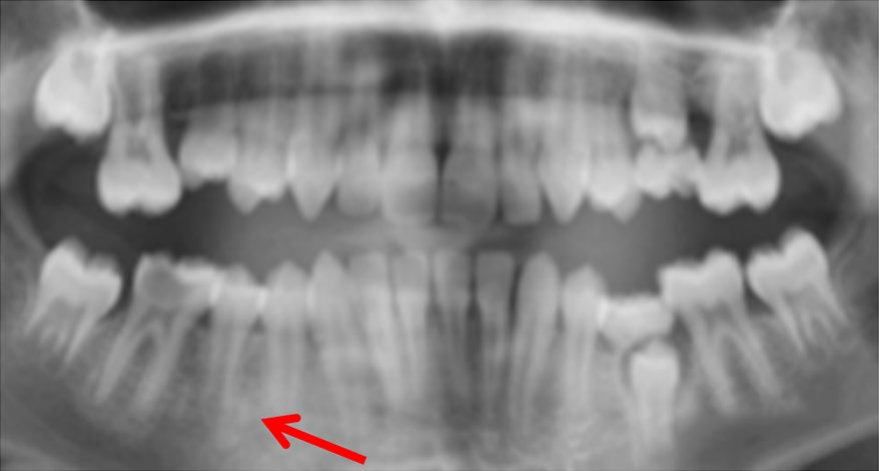
Dental panoramic tomographs showing DBIs (see arrows) on the mandibular right quadrants, mesial to LR5 (case 13)

**Fig. 14:**
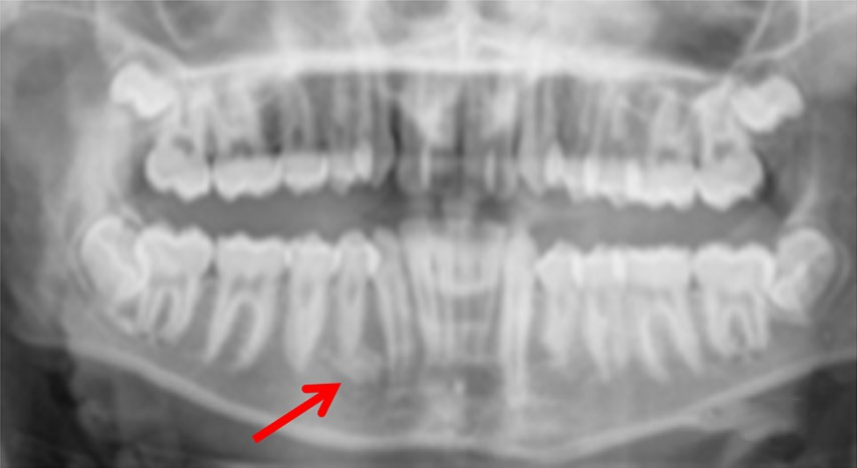
Dental panoramic tomographs showing DBIs (see arrows) on the mandibular right quadrants, apical to LR4 (case 14)

Case 10 was referred for specialist radiology opinion following mid-orthodontic treatment in primary care. Resorption of the mesial root of LR6 was noted in close proximity to a dense bone island (Fig. 10a, b). Monitoring of LR6 was advised (Figs. 11, 12, 13 and 14).

**Fig. 15:**
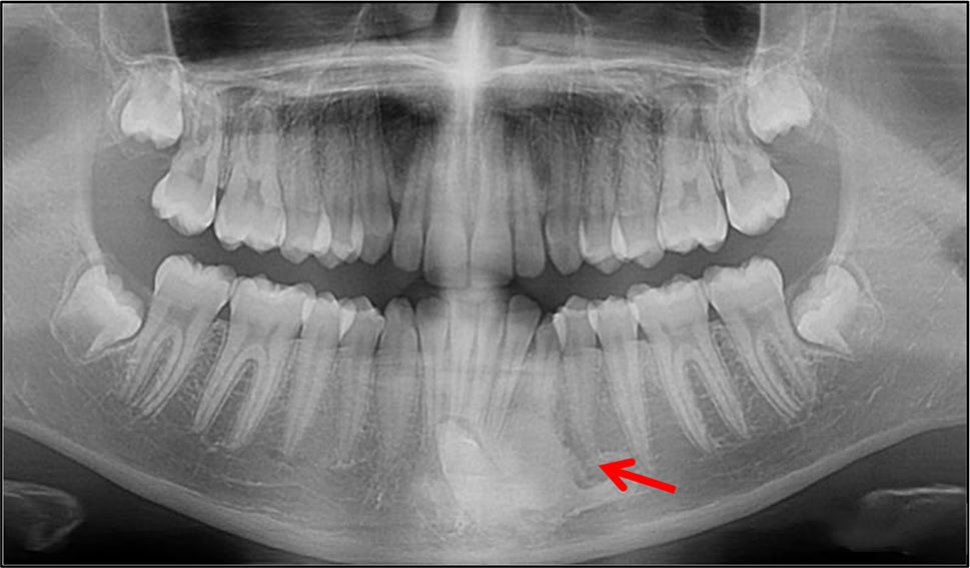
Dental panoramic tomograph showing a dense bone island on the mandibular left quadrant associated with the ectopic, unerupted LL3

Case 15 (Fig. 15) had an extensive bone island that was probably responsible for the ectopic position of the LL3. Following multidisciplinary discussion, it was decided to extract LL3 and monitor the dense bone island.

**Fig. 16:**
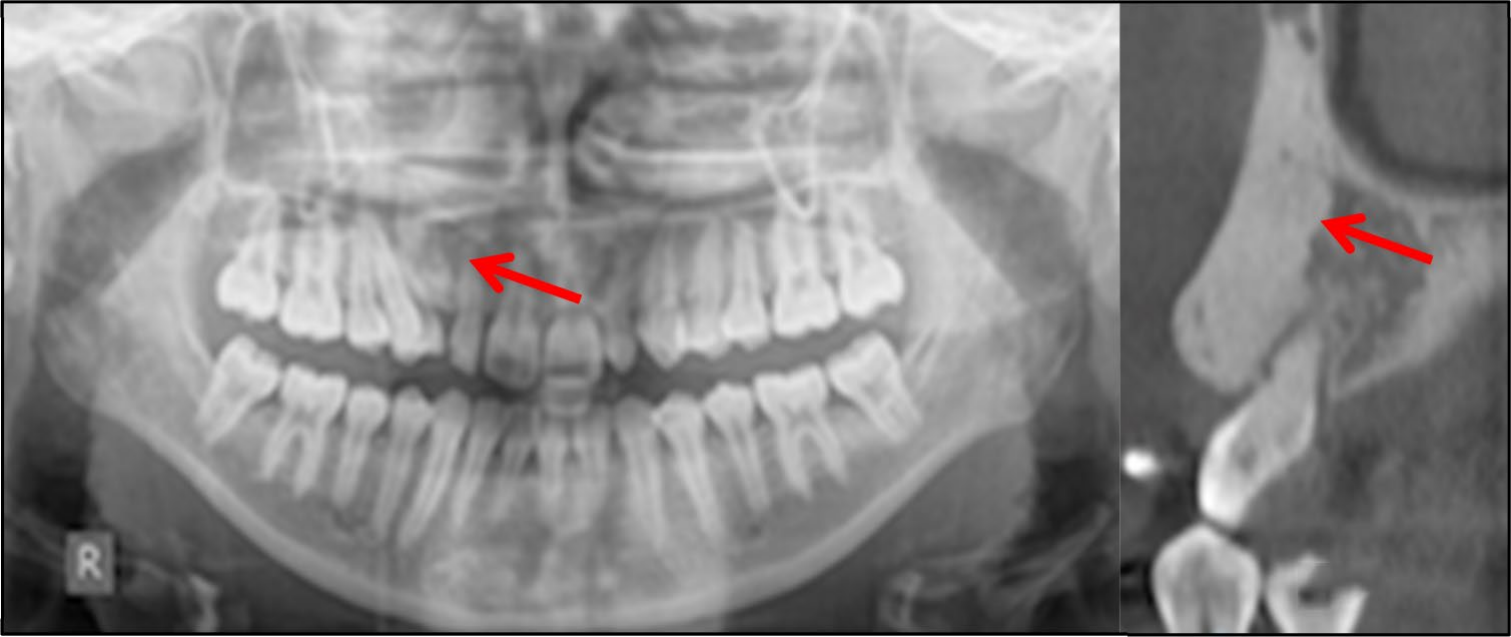
Dental panoramic tomograph and cone beam computed tomography showing a large dense bone island on the upper right quadrant between UR3 and UR2

Case 16 (Fig. 16) had a large bone island on the buccal aspect of the maxillary alveolar process, occupying at least 50% of the alveolar process between UR3 and UR2 and extending to the right nasal fossa. In this case, following multidisciplinary discussion, orthodontic treatment was deemed contra-indicated in the area of the dense bone island.

Diagnoses were confirmed by a Dental and Maxillofacial Radiology Consultant and advised no intervention. In some cases, monitoring was advised. Caution in orthodontic tooth movement was advised for five patients (cases 2, 3 5, 12 and 16), with orthodontic tooth movement in the area of the dense bone island being contraindicated due to increased risk of resorption of adjacent tooth roots.

## Discussion

Most DBI lesions are discovered incidentally during routine radiographic examination. The size of the lesions is usually stable, with rare reports discussing potential for enlargement (Greenspan and Stadalnik 1995; Petrikowski and Peters 1997; Mariani et al. 2008). Nakano et al. in 2002 found a 7% increase in size of a DBI on a 10-year-old girl. This is in agreement with Petrikowski and Peters’ conclusion for children and teenagers that about 40% of DBIs are thought to increase in size a period of 10 years and about 17% decrease in size for the same period. It is thought that this increase in size accompanies normal bone growth. In our case series, we found no size changes during our follow-up times. In the absence of agreed guidelines, the authors suggest adoption other authors’ advice that the lesion is monitored at least until the patient’s growth is completed and the lesion has stopped evolving (Nakano et al. 2002).

Although DBIs are often of no clinical significance, dental extraction of a tooth embedded into a DBI may result in an infected socket and pain of the edentulous area (Marmary and Kutiner 1986). In this case series, however, none of these findings was noted as no extractions were undertaken in close proximity to a DBI. We found one case report of an implant placed in close proximity to a dense bone island. The authors resourced to CBCT imaging and advised extreme care in planning as well as increased period of follow-up post operatively (Li et al. 2016).

Two case reports found symptoms related to dense bone islands present in the femur and in the tibia of a 10 and a 69-year-old, respectively. In these cases, DBIs were managed with excision followed by histology (Greenspan et al. 1991; Diab et al. 2014). One paper report on neuropathy related with compression of the inferior dental nerve due to the presence of a DBI (Debevc et al. 2017). Although none of our patients reported any DBI-related symptoms, the authors recommend timely diagnosis and accurate treatment planning for DBIs.

Medically, DBIs are of little clinical significance; however, a differential diagnosis should be established to rule out osteoblastic metastases from a known primary tumor (Nakano et al. 2002). Although DBIs are histologically different to osteomas, it is important to consider these as differential diagnoses as these may be associated with adenomatous intestinal polyps which may suffer malignant transformations (Butler et al. 2005, Sinnott and Hodges 2020). Multiple DBIs, with or without associated osteomas, may be a feature of Gardner’s syndrome (Davies 1970). Suspected medical findings must be discussed with the patient’s medical practitioner.

This case series reports sixteen cases of DBIs. All cases required multidisciplinary management, with radiology assessment and reporting for diagnosis. Three patients had initiated or completed orthodontic treatment prior to diagnosis of DBI, two with no unfavorable outcomes to adjacent teeth. In other cases, caution was advised for orthodontic tooth movement in the proximity of the DBI. Liaison with orthodontic colleagues was undertaken in these cases. The remainder of patients required no further investigations or treatment, other than monitoring. However, one patient (case 10) was referred for an opinion mid-treatment, after root resorption had occurred. The presence of DBIs offers additional challenges for orthodontic treatment, including difficulty with achieving space closure and adequate root tip or torque (Sinnott and Hodges 2020). Due to these challenges, and the possibility of iatrogenic root resorption in relation to a DBI, care is recommended when planning orthodontic interventions.

Sinnott and Hodges (2020) suggest that CBCT may be beneficial to ascertain the full extent of the lesion. In this case report, a DBI seemingly measuring 6 mm on a periapical radiograph was found to be significantly larger (24 mm). In our case series, two patients were found to benefit from CBCT prior to orthodontic planning.

**Fig. 17:**
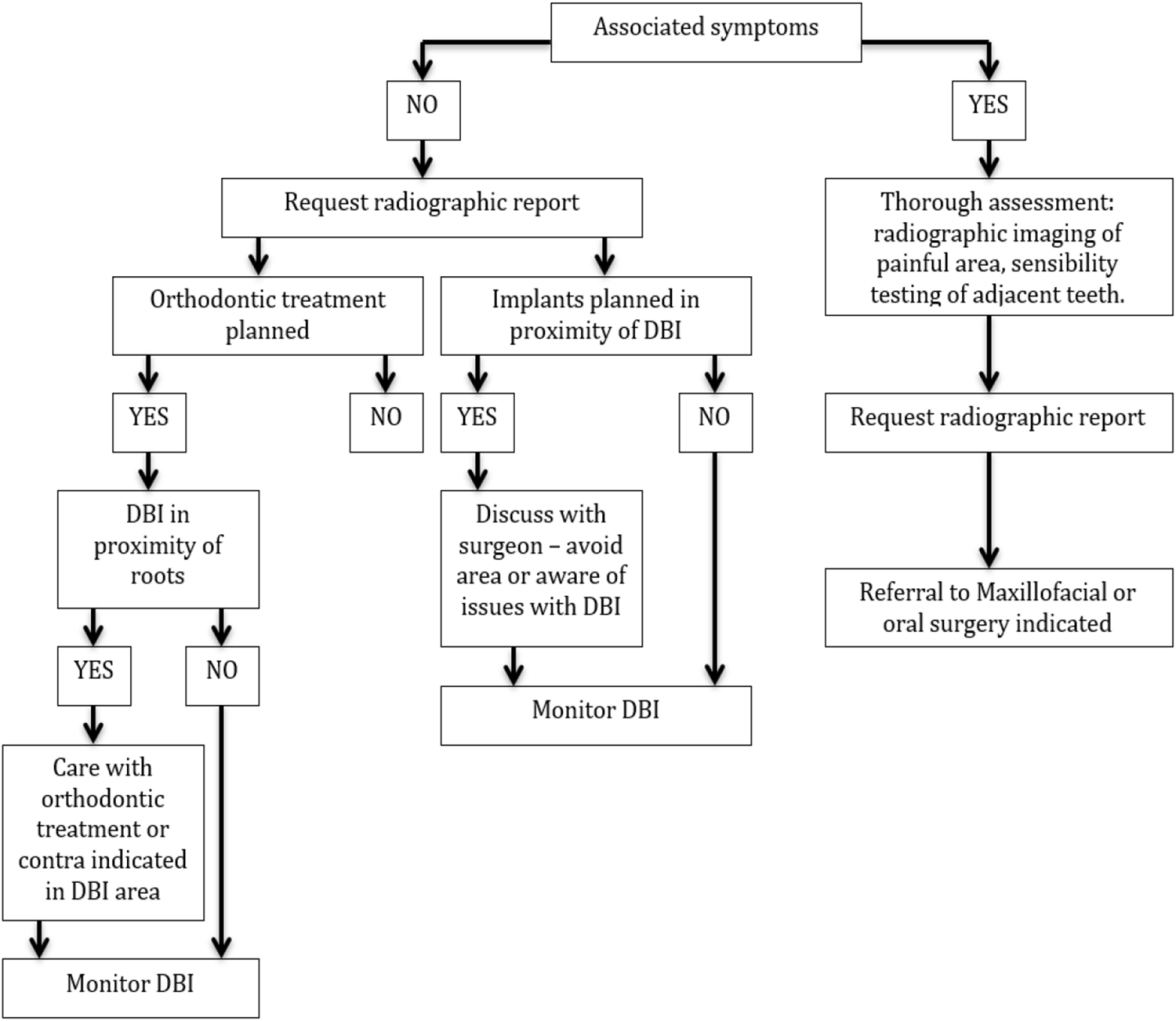
DBIs management flowchart

Following revision of the literature and findings from our case series, a management flowchart is proposed to aid in treatment planning of DBIs (Fig. 17). These findings include the need to monitor the lesion and request radiographic report when required. In addition, if orthodontic treatment or implant placement is required, care must be taken especially if the DBI is in close proximity to the roots or implant.

## Conclusion

DBIs are common findings that seldom require treatment; however, caution should be exercised when undertaking orthodontic movement in the area of a DBI due to a potential risk of root resorption. Accurate identification and multidisciplinary management are of utmost importance. Monitoring size changes is recommended until completion of patient’s growth.
